# Geometric Shape Induced Small Change of Seebeck Coefficient in Bulky Metallic Wires

**DOI:** 10.3390/s17020331

**Published:** 2017-02-10

**Authors:** Gang Li, Xiaohui Su, Fan Yang, Xiaoye Huo, Gengmin Zhang, Shengyong Xu

**Affiliations:** Key Laboratory for the Physics & Chemistry of Nanodevices, and Department of Electronics, Peking University, Beijing 100871, China; pkloylee@pku.edu.cn (G.L.); suxiaohui211@163.com (X.S.); fyang1992@pku.edu.cn (F.Y.); xiaoye.huo@tx.technion.ac.il (X.H.); zgmin@pku.edu.cn (G.Z.)

**Keywords:** thermopower, bulky metallic wire, mean free path, size effect, high-temperature sensor

## Abstract

In this paper, we report the results of slight changes in the thermopower of long W, Mo, Zn, Cu, brass, and Ti wires, that resulted from changes in the wire’s diameter or cross-sectional area. The samples used in the tests had a round shape with a diameter that ranged from tens of micron to 2 mm, which was much larger than the corresponding mean free paths of these materials. Nevertheless, a small change in thermopower, at the order of 1–10 nV/K, was repeatedly observed when the wire diameter was changed, or when the cross-sectional area of the wire was altered by mechanical methods, such as grinding or splitting. The results are consistent with previous observations showing that the thermopower in metallic thin film stripes changes with their width, from 100 μm to as little as 70 nm, implying a universal, geometric-boundary-related size effect of thermopower in metal materials, that occurs at the nanometer scale and continuously decreases all the way to the millimeter scale. This effect could be applied in the manufacturing of high-temperature sensors with simple structures.

## 1. Introduction

Metals and metallic alloys are important functional materials for modern devices and electronic systems. As high-strength materials, they have been extensively applied as frameworks and covers for most manmade products. As excellent electrical conductors, metallic wires are used as power lines, and Al, Cu, and W thin films are applied as interconnects in most conventional electronic circuits and in state-of-the-art integrated circuits [1,2]. Au, Ag, Pt, Cu, and Al thin films are intensively applied as electrodes in lab-on-a-chip systems [3], micro-electro-mechanical systems (MEMS) [4], and nano-electro-mechanical systems (NEMS) [5], as well as in a variety of quantum electronic devices [6,7,8,9,10], and novel medical devices embedded in the human body [11,12,13,14]. On the other hand, by using the thermoelectric properties of metallic thin films, many kinds of micro-scale and nano-scale thermal sensors have been developed from a variety of metallic thin films [15,16,17,18,19,20,21,22]. It is believed that metallic thin-film-based sensors should play an important role in the next generation of flexible and wearable electronics.

A better understanding of the metallic materials is helpful for the development of modern devices and systems. It is known that, when the thickness of a metallic thin film is less than 100 nm, which is comparable to the mean free path of electrons in a metal, a strong thickness-dependent effect occurs in the scattering processes of electron-electron and electron-phonon interactions, resulting in a drastic reduction of conductivity when the film thickness further decreases [23]. This effect becomes one of the critical issues in design and fabrication of interconnects in the latest large-scale integrated circuits [1]. Similarly, such an effect occurs in the thermoelectric power of the film [24,25,26,27,28,29,30,31]. A voltage signal can be observed when a temperature gradient is set up between the hot and cold end of the thermoelectric materials. Often referred to as the *Seebeck coefficient*, the thermopower *S* is defined as *S* = Δ*V/*Δ*T*, where Δ*V* is the voltage difference established between the two ends of a metal or semiconductor material, and Δ*T* is the temperature difference [32]. In metals, *S* is sensitive to the density of states (DOS) of the charge carriers near the Fermi surface. It is usually described with the Mott-Jones equation [33,34]:

(1) $$S=-\frac{\pi^{2}k_{B}^{2}T}{3e}{\left(\frac{d\ln\sigma }{dE}\right)}_{E=E_{F}}=-\frac{\pi^{2}k_{B}^{2}T}{3e}{\left(\frac{d\ln\lambda }{dE}\right)}_{E=E_{F}}-\frac{\pi^{2}k_{B}^{2}T}{3e}{\left(\frac{d\ln A}{dE}\right)}_{E=E_{F}}$$

where *S*, *k_B_*, *T*, *e*, *σ*, *E*, and *E_F_* are the thermopower, Boltzmann constant, temperature, electron charge, electrical conductivity, electron energy, and the Fermi energy, respectively; *λ* is the mean free path of electrons and *A* is the area of the Fermi surface. Here, the electrical conductivity *σ* is expressed as *σ* = *e*^2^*λA*/6*π*^2^*h*. When the thickness of a metallic film is more than 100 nm, which is larger than the *λ* of most metals at 300 K, it is generally predicted that the Seebeck coefficient should approach a stable value [24,25,26,27,28,29,30,31]. 

However, exceptions to the predictions of mean field theories have been repeatedly reported, and the thickness dependence of electrical transport and thermoelectricity has remained an interesting topic over the years. In experiments with gold, silver, and copper thin films, Leonard et al. showed that a change in the thermopower occurred at a film thickness of around 250 nm, which was five to six times the mean free path of these metals [35,36,37]. Similar phenomena were found in ytterbium, samarium, tin, palladium, and chromium thin films by Angadi et al., at a film thickness of less than 200 nm [24,25,26], and in thin films of aluminum [27], bismuth [28], iron [29], and other metals [30,31]. Most of these results were in good agreement with the Fuchs-Sondheimer model (FS model) [33,34]. Fuchs and Sondheimer discussed the surface scattering mechanism of electron transport in metallic thin films, and introduced a specular scattering coefficient, *p*, as the fraction of electrons being scattered. Their work results in a thermopower *S_f_*, expressed as:

(2) $$\frac{S_{f}}{S_{bulk}}=1+\frac{3}{8}(1-p)\frac{\lambda }{t}\frac{U}{1+U},\;U={\left(\frac{d\ln\lambda }{dE}\right)}_{E=E_{F}}.$$

where *λ* and *t* are the intrinsic mean free path and film thickness, respectively. Soffer [38] further developed this *p*-parameter analysis and related the change in *S_f_* to the surface roughness. Generally, Equation (1) leads to a rough relation of (*S_f_* − *S_bulk_*) ∝ 1/*t*. This well-developed theory has been successfully applied to the description of experimental data on the thickness dependence of the thermopower of a variety of metallic films [23,24,25,26], as well as the results of enhanced *ZT* in patterned Si nanowires [39].

Recently, for the metallic thin film stripes of more than ten metals, such as Ni, Cr, Ti, Ta, Pd, and W, it was reported that, when the film thickness was kept at 80–100 nm, but the stripe width was changed from 100 micron to 30–50 μm, the thermopower of these stripes continuously showed measurable changes, and the size effect was enhanced when the stripe width was further reduced, all the way to 80 nm [19,40,41,42,43]. This phenomenon was found following a brief power law of *w*^−*α*^, where *w* was the stripe width and *α* was a number close to one [41]. Since the length of 30–50 μm is two orders of magnitude larger than the mean free paths of the ordinary bulk metals tested in these studies, the results raised arguments [44,45]. However, this study raised two interesting questions: What is the underlying mechanism? And, could such a phenomenon be observed in even thicker materials?

In this paper, we report our experimental results for the thermopower measured from thick metallic wires with diameters ranging from 70 micron to 2–3 mm, which were presumably considered to be “bulky” wires. Surprisingly, in the “bulky wires” of W, Mo, Cu, brass (CuZn), Zn, and Ti, slight but unambiguous reduction changes in the thermopower are measured within the temperature range of 300–1500 K, when the cross-sectional area or diameter of these wires were altered. We believe that the various geometric scales induced the slight change in the *Seebeck coefficient* of homogenous thermoelements. The results indicate a universal size effect of thermopower in metal materials that occurs at the nanoscale, and decreases, but continues to exist, from the nanoscale up to the millimeter scale.

## 2. Experimental Details

In this work, we have made samples from dozens of metallic wires with diameters ranging from 70 μm to 2–3 mm. The main ingredients of these wire materials, offered by the commercial suppliers, are listed in Table 1. 

### 2.1. Preparation of Type-U Samples

**Figure 1:**
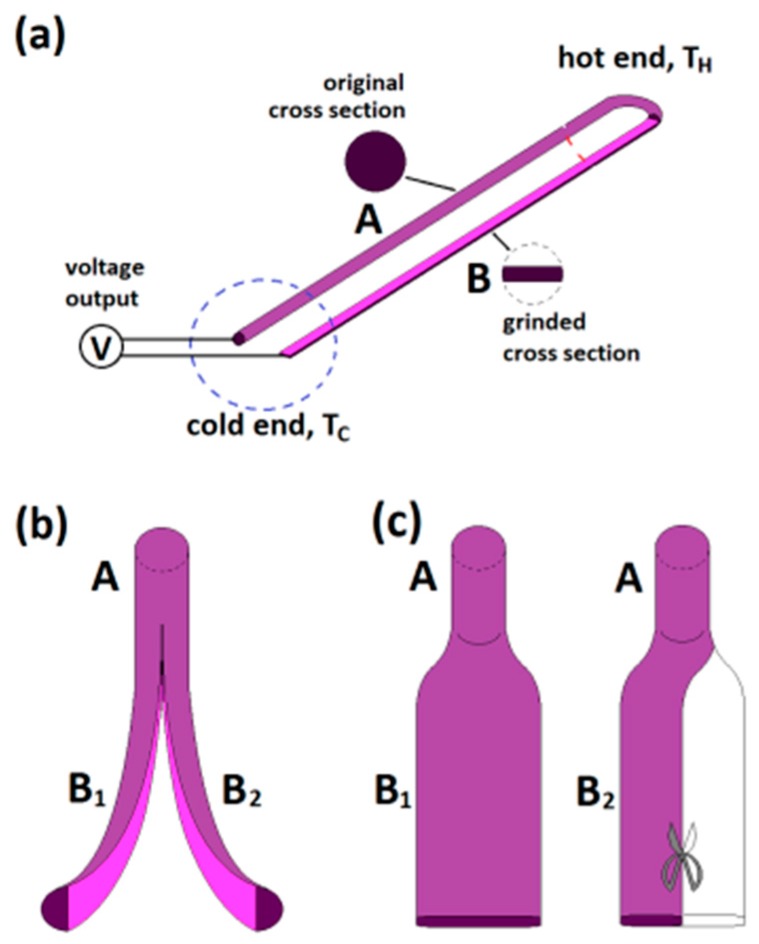
Schematic figures for the sample preparation: (**a**) A “Type U” sample made from one piece of thick wire, where one arm (marked “**A**”) is in its original round cross-section shape and the other one (marked “**B**”) is mechanically thinned to have a thickness of ¼–½ of the original diameter; (**b**) A split sample, which is always made from one piece of thick W wire; (**c**) A pressed sample, where the arm “**B_1_**” is mechanically pressed to be much thinner than the diameter of the original wire “**A**”, and the thin arm “**B_1_**” of such a sample could be further cut into narrower arm “**B_2_**” for a second run measurement.

For most of the samples used in this work, each individual sample was made from one piece of long wire of a specific material, and these wires had a diameter which ranged from 0.5–2.0 mm. In each sample, part of the wire was kept in its original shape, and the rest of it was grinded, split, or pressed, so its cross-sectional area was reduced to ¼–½ of the original wire. For the soft wires of Cu, brass (Cu-Zn), Zn, Ti, Mo, and W, each sample was bent into a “U” shape, where the thick arm of the sample was kept as the original wire diameter, but the thin arm was thinned with an electrical grinding wheel, or mechanically with a grinder and sand paper. Figure 1a shows such a sample configuration. The turning part of the sample serves as the junction region or the “hot end” in the following measurement process. For hard W wires, bending over 180° usually leads to a broken bending section. So, we kept W wire samples straight in shape, but half of the long wire of each sample was mechanically thinned using a grinder, and the testing hot end was chosen at the joint part of the thin and thick arms.

For the remainder of this paper, both the “U” shape and straight shape dual-arm samples are referred to as “Type U” samples. The merit of this “U” shape configuration is that, at the nominal junction region, there is no interface between the two arms, because they originally belong to the same piece of long wire. This is the main reason that we could detect a tiny difference in thermopower between these two arms, to the relative level of 10^−4^ of the bulk value.

In the process of grinding Mo and W wires, which were very hard, the temperature of the sample significantly increased. At such a high temperature, the supporting wood plate of the wire got burnt, which implied that the temperature was more than 500 K. This could induce slight changes in the crystalline structure of the wire, and consequently, a cooling process of the heated wire underwent an annealing process. 

In order to avoid the heating and annealing effects in the preparation stages, we fabricated samples with various hand grinders for Cu and brass (CuZn) wires, which were much softer than Mo and W. In each of these samples, the thick arm kept its original diameter, 1.0–2.0 mm, and the thinner one, which was hand-grinded, had a cross section area of one fourth to a half of the original value. 

### 2.2. Preparation of Split Samples

The splitting nature of the pure W wire was applied in the preparation. A set of split samples were made from W wires and measured. In most cases, it was easy to split a short piece of W wire (diameter 0.5–1.0 mm) into two halves with a short length, each having similar size. But, it is very hard to obtain long split samples. The sample configuration is shown in Figure 1b. 

### 2.3. Preparation of Pressed Samples

Since Zn wires are very soft, we prepared one sample by directly pressing a 1.5-mm diameter, 2-m long Zn wire, so that 1 m of the wire maintained its original shape, and a further 1 m changed its round cross sectional shape to a rectangular shape, causing it to become wider, as shown in Figure 1c. This sample was tested first, and then the pressed section was mechanically cut in half, width-wise, for a second run testing. To ensure the repeatability of the experiments, dozens of Mo, Ti, W, Cu, brass, and Zn wires were examined. These wires had an original diameter which ranged from 0.08 mm (80 μm) to 2.0 mm. This dimension range is two to four orders of magnitude higher than the critical lengths of electrons or phonons in these metals. 

### 2.4. Preparation of Thick-Wire, Thin-Wire Dual-Arm Samples

In addition, tens of Mo, W, and Ti wire samples were simply made with two individual pieces of commercial pure wire, with diameters that varied from 0.07 mm to 1.0 mm. At the hot end, two wires of the same sample were mechanically jointed. These samples were tested as potential sensors in practical high-temperature measurements.

To obtain reliable measurement data, the samples were tested in three different heating systems. Two of them were commercial ovens with maximum temperatures of 1000 K and 1500 K. The third system was a home-made system, which consisted of one sample, one type-K thermocouple, several layers of ceramic fiber paper (aluminum silicate) as insulating layers, and two W plates as the cover and bottom of the device. These components were packed together with fixing screws and W wires. The hot end of the thermocouple was located as close as possible to the joint of the thick and thin arm of each sample, but was electrically insulated from the metallic wire sample being tested. During each measurement, the testing device was fixed on a solid frame over an alcohol burner, with a spacing of 10–15 cm, where the flame of the alcohol burner produced a maximum temperature of around 700 K at the sample’s hot end. 

For all measurements, the voltage outputs of the samples, measured at the two cold ends, were recorded with a Keithley 2182 Nanovoltmeter. Suppose the thermopower values of the two arms (marked as **A** and **B** in Figure 1) are *S*_A_ and *S*_B_, respectively. Define Δ*S* = |*S*_A_ − *S*_B_|. If *S*_A_ ≠ *S*_B_, then a net voltage Δ*V* will be measured at the cold ends, Δ*V* = |*S*_A_·Δ*T* − *S*_B_·Δ*T* | = |*S*_A_ − *S*_B_|·Δ*T =* Δ*S*·Δ*T* > 0. In each measurement, the cold ends of a sample were located far away from the heating zone, as all of the samples were as long as 0.4–3.0 m. To avoid any possible thermal conductance influence on the measurement results, both arms of each sample were separately cooled in a sequence of several plastic water tanks, located between the hot and cold ends. The water temperature of each water tank was kept as room temperature, by changing replacing the contents with fresh water during the measurement. In some experiments, we used iced water to replace the room temperature water, and the results remained the same. For each sample, measurements were performed repeatedly for several cycles of heating and cooling processes. For some samples, the same sample was measured again after a long period of time (from a few days up to one year), in order to check the reliability.

As a calibration of the system setups, control samples of identical wire couples were measured. Hereinafter, the “identical wire” sample refers to a “Type-U” sample made of a single wire without any treatment, where the two arms of the sample are identical, and the heating zone (hot end) is set at the turning point of the wire.

## 3. Results

### 3.1. Results of Grinded Type-U and Split Samples

**Figure 2:**
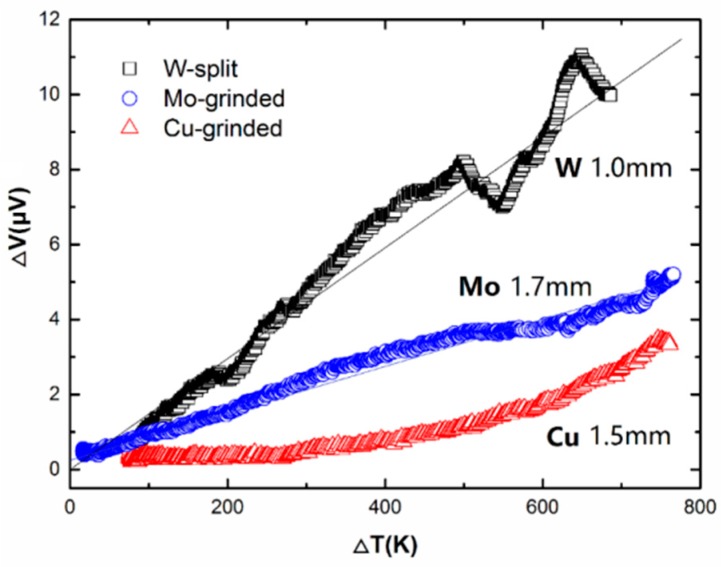
Measurement results of the voltage at the cold ends versus temperature for one splitting W sample (original diameter 1.0 mm), one grinded Mo sample (original diameter 1.7 mm), and one Cu sample (original diameter 1.5 mm).

Figure 2 shows the typical measurement results of a grinded Mo sample, a grinded Cu sample, and a split W sample, in which only the data associated with the heating processes were plotted. The original wire diameters of the Mo, Cu, and W samples were 1.7 mm, 1.5 mm, and 1.0 mm, respectively. For these samples, the cross-section shapes of the grinded arms and split arm were all a semi-circle, and the cross-sectional area of each sample was roughly half that of the original wire. The length of these samples was longer than 1 m.

By rough linear fitting, the slopes for the data of the Mo sample and the W sample produce slopes (i.e., Δ*S*) of 0.006 μV/K and 0.015 μV/K, respectively. The slope for the data of the Cu sample is close to zero when Δ*T* is small, and increases to 0.010 μV/K at Δ*T* > 600 K, with an average Δ*S* of ~0.005 μV/K.

It is interesting to determine the dominant factor that leads to the small change in thermal power, and it is also interesting to investigate whether such a phenomenon is universal in metals. To rule out factors other than the geometric effect, we prepared samples made from a single piece of long wire, where one arm was thinned from both sides with an electrical grinding wheel. 

**Figure 3:**
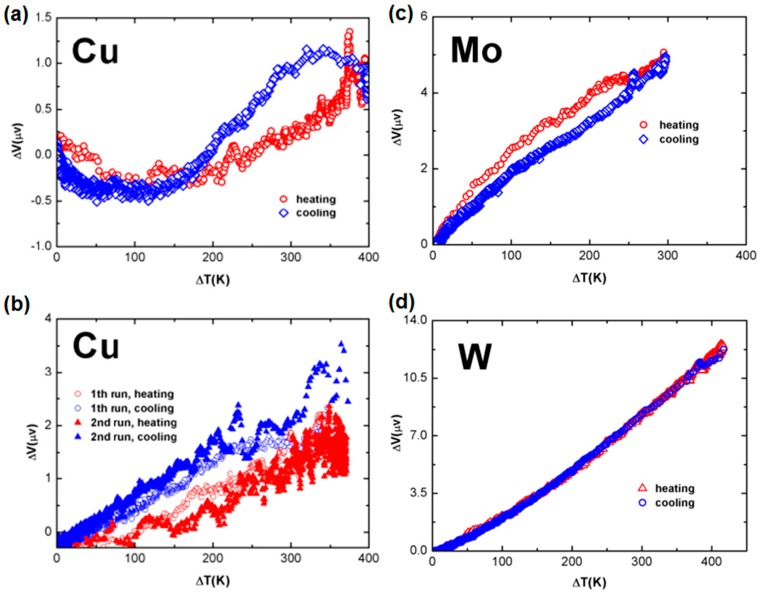
Measurement results of grinded samples made from (**a**) a 2.0 mm diameter pure Cu wire sample; (**b**) a 1.0 mm diameter pure Cu wire sample; (**c**) a 0.5 mm diameter pure Mo wire; and (**d**) a 0.5 mm diameter W wire. For all of these samples, the thinned arm has a cross-sectional area which is about half of the original wire’s cross-sectional area.

Figure 3a presents the measurement results of a sample made from a long, 2.0 mm diameter pure Cu wire. The length of each arm was 45 cm. The cross sectional area ratio of the thick arm to the thin arm, was about 2.0. The maximum change in Δ*V* at Δ*T* = 350 K is measured to be around 1.2 μV, which is barely larger than the noise level. However, in samples made from thinner pure Cu wires, the measured signals were bigger. Figure 3b shows the data of the two runs measured for a pure Cu wire sample, with an original diameter of 1.0 mm. The cross sectional area ratio of the thick arm to the thin one, was around 2.0, and the arm length was 55 cm. When studying the cooling curves, one notes that Δ*V* reaches 3.0 μV at Δ*T* = 350 K. This is more than twice of that measured in the 2.0 mm Cu wire sample (Figure 3a). 

Figure 3c plots the measurement results of a Mo sample. Each arm of this sample was 37 cm long. The original wire was a 0.5 mm diameter pure Mo wire, with a round cross sectional shape, but the grinded arm had a rectangular cross sectional shape, with an average area of 0.5 mm × 0.15 mm. The curve is almost linear, where Δ*V* is around 5.0 μV at Δ*T* = 300 K, leading to a slope of about 0.016 μV/K. This is a very small change that could be overlooked in most of the measurement systems. Figure 3d shows the results of another electrically grinded sample, made of a pure 0.5 mm diameter W wire. The arm length was 38 cm. The grinded arm also had a rectangular cross sectional shape, with a cross section which had an average area of 0.5 mm × 0.2 mm. The measured data was repeated for both the heating and cooling processes, where Δ*V* reaches 12.0 μV at Δ*T* = 400 K, showing a slope of 0.03 μV /K, roughly twice that of the grinded Mo sample. 

**Figure 4:**
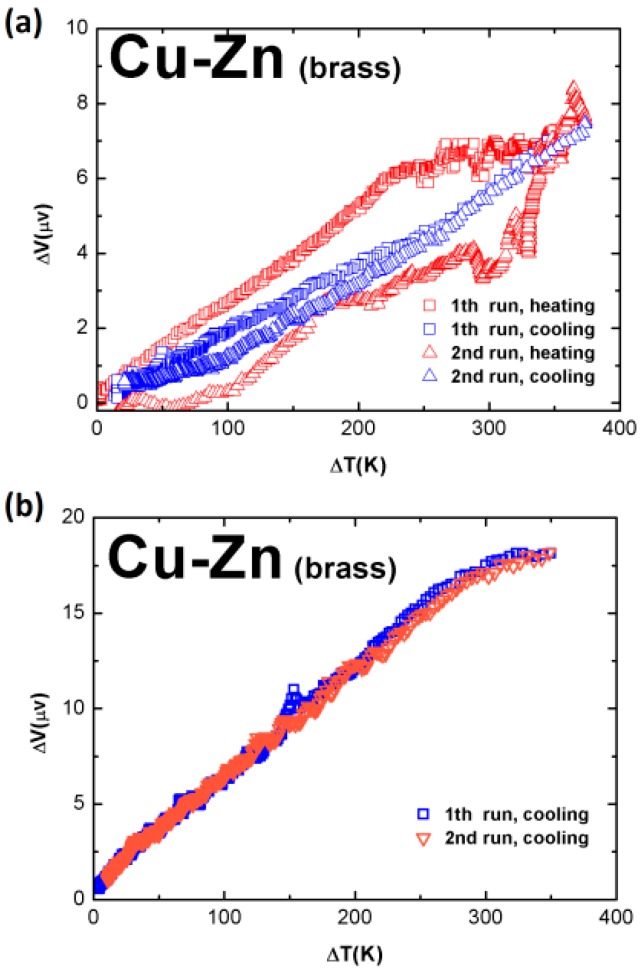
Measurement results of brass wire samples: (**a**) A sample with an original diameter of 2.0 mm; and (**b**) a sample with an original diameter of 1.0 mm. The cross sectional area ratio of the thick arm to the thin, for both samples, is around 3.0.

Larger signals were obtained from samples made of brass wire. Figure 4a plots the data measured during the second runs of the heating and cooling processes of a brass wire sample, with an original diameter of 2.0 mm. For both samples, the cross sectional area ratio of the thick arm to the thin, was around 3.0, and the length of each arms was 58 cm. In this sample, Δ*V* is about 7.0 μV at Δ*T* = 350 K. In Figure 4b, one sees the experimental data measured during the cooling process of a thinner brass wire sample, with an original diameter of 1.0 mm. For both samples, the cross sectional area ratio of the thick arm to the thin, was about 2.0, and the length of each arm was 44 cm. In this sample, Δ*V* becomes 17.5 μV at Δ*T* = 350 K.

### 3.2. Results of Pressed Samples

**Figure 5:**
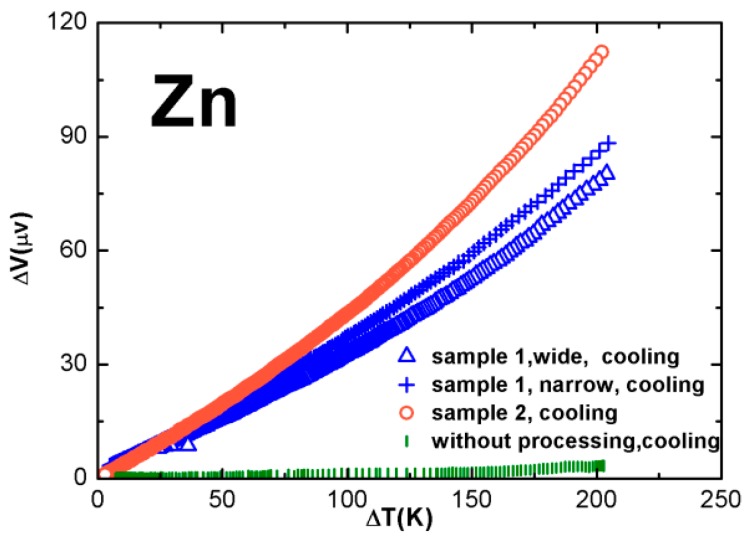
Measurement results of pressed samples made from one piece of 1.5 mm diameter Z wire.

Figure 5 plots the experimental results obtained from two Zn wire samples. The thin arms of these samples were not grinded, but mechanically pressed with a hammer. The cross sectional shape of the original wire was round, with a diameter of 1.5 mm, and that for the pressed arm was flat and rectangular. During the pressing process, the pressed arm was extended slightly in the longitudinal direction, and the final cross sectional area of the pressed flat arm was smaller than that of the original round wire, by 20%–30%. As shown in Figure 5, the arm length of “sample 1” was 24 cm, and that for “sample 2” was 57 cm. The one marked by “sample 1, narrow” was made from “sample 1, wide”, by cutting away half of the pressed flat arm along the wire; therefore, it reduced the cross sectional area of the remaining arm by two. The ratio of the cross sectional area of the original sample to the pressed ones, was 1.2–1.3 for both “sample 1, wide” and “sample 2”. The ratio for “sample 1, narrow” was about 2.5. 

In Figure 5a, it is possible to view the trend that, as the ratio of the cross sectional area of the thick arm to the thin increases, the measured change in the *Seebeck coefficient* Δ*S* monotonously increases. The measurements were limited to Δ*T* = 205 K, to avoid melting the wires, and at this temperature, Δ*V* values were 80, 88, and 112 μV, respectively, for these three samples, corresponding to Δ*S* of 0.37–0.49 μV/K.

### 3.3. Calibration Results of “Identical Wire” Samples

**Figure 6:**
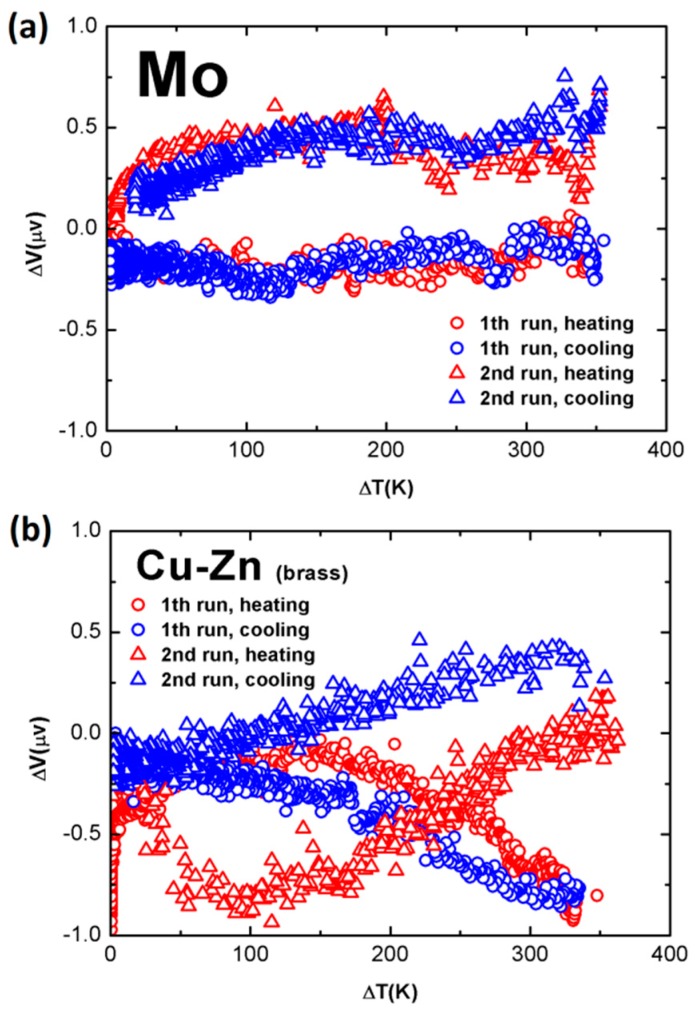
Calibration results of “identical wire” samples made from (**a**) one piece of 0.5 mm diameter Mo wire and (**b**) one piece of 1.0 mm diameter brass wire.

Presumably, the voltage signals measured from the “identical wire” samples were expected to be within the noise level of the whole measurement system. This was true for most of the wires tested in this work. To avoid any possible thermal conductance, both arms of the sample were separately cooled in several plastic water tanks, located between the hot and cold ends. The water temperature of each tank was kept at room temperature, by changing the water during the measurement. Figure 6a presents a typical result, which was measured from a 0.5 mm diameter, 1 m long Mo wire. The sample was measured four times. For the first run, the sample was tested during a heating process, where the temperature difference between the hot and cold ends was increased from zero to 350 K, with an alcohol burner. Then, the sample was tested immediately during the cooling process, where the alcohol burner was removed. The data are plotted in triangles for these two processes. For the second run, the measurement electrodes were exchanged, and the whole measurement procedure was repeated from Δ*T* ≈ 0 K. This step changed the sign of the dc voltage signals. If the measured signals were system noises, the data were expected to show similar trends to those of the first run.

In Figure 6a, one sees that, for each run, the signals measured during the heating process are in good agreement with those recorded during the cooling process. Additionally, at the same Δ*T*, the absolute difference in the signals between the two runs is mostly within 0.8 μV, over the entire temperature range. This indicates that the whole set of measurement data are within the noise level of the current measurement setup, as expected.

Figure 6b presents a typical result measured from an “identical wire” couple of a 1.0 mm diameter, 1.0 m long brass wire. Here, the same pattern is shown as in Figure 6a, and the measurement electrodes were exchanged for the second run. The difference gradually increased with increasing Δ*T*, and at Δ*T* = 330 K, Δ*V* was around 1.0 μV, which we considered as a system error. 

### 3.4. Prototype High-Temperature Sensors, Thick-Wire, Thin-Wire Dual-Arm Samples

Although the change in thermopower in a bulky metallic wire, due to the change in its cross-sectional area, is very small, i.e., Δ*S* ~ 1–10 nV/K, this phenomenon may have application in high-temperature sensors. When Δ*T* is approaching 1000–2000 K, a small Δ*S* can still lead to a measurable Δ*V* as Δ*V* = Δ*S*·Δ*T*. That is, the output voltage has been enhanced by three orders of magnitude.

**Figure 7:**
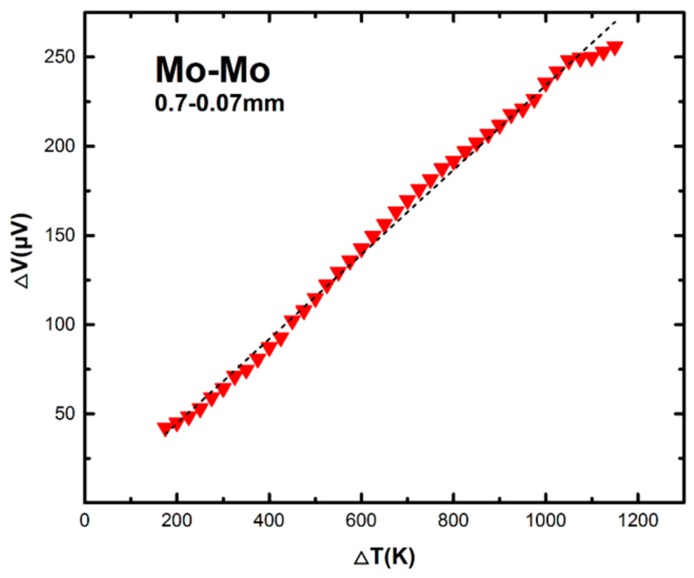
Measurement results for a sample made for two pieces of Mo wires with diameters of 0.07 mm and 0.7 mm, respectively. It shows a roughly linear response in the voltage output over a large temperature difference between the hot and cold ends up to 1200 K.

Some Mo and W samples, each made of a thin wire and a thick wire, were tested in air as a prototype “sensor”, for a high temperature difference up to 1500 K. Such a device structure is probably the simplest to construct. Figure 7 presents a typical result for a Mo sample, which is made from two pieces of Mo wires with diameters of 0.07 mm and 0.7 mm, respectively. The whole heating process takes 35 min. The reading of the oven temperature is made with a standard commercial Pt-Rh thermocouple. The experimental data are close to a linear response to the increasing temperature. The linear fitting line produces a slope of 0.236 μV/K.

**Figure 8:**
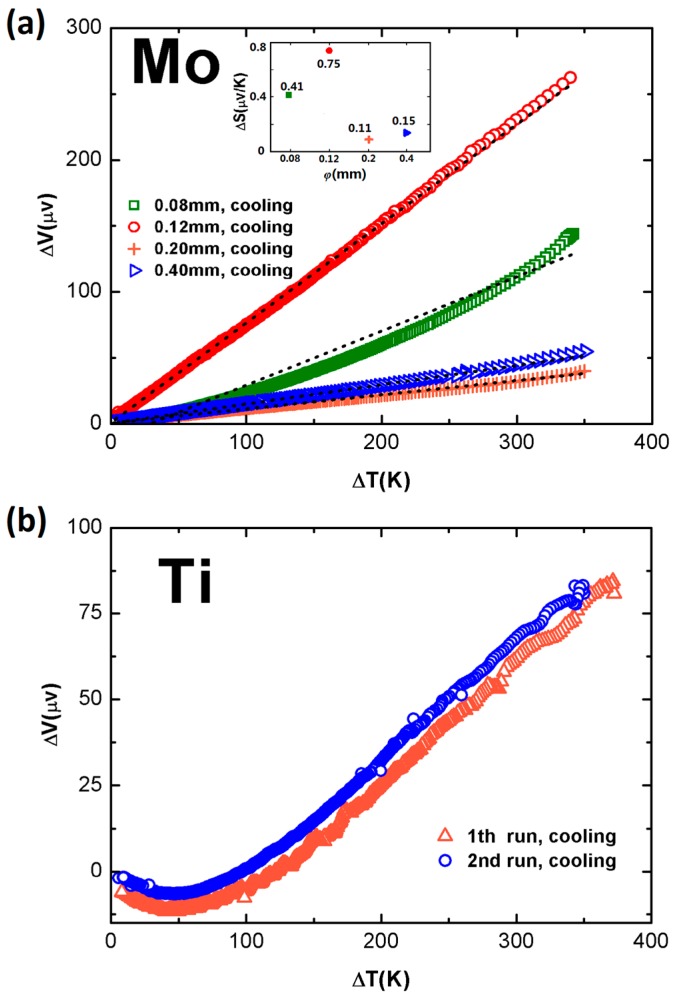
Measurement results for a bunch of thick-wire, thin-wire dual-arm samples made from (**a**) Mo wires; and (**b**) Ti wires.

A variety of thick-wire thin-wire dual-arm samples were quickly measured with our homemade testing device. Figure 8a plots the experimental results of a group of pure Mo-Mo samples. Each sample was made of a pair of 1 m long thick and thin Mo wires. The thick wires had a fixed diameter of 0.7 mm, and the thin wires had a diameter of 0.08, 0.12, 0.20, and 0.40 mm, respectively. Each sample was measured over several runs. To avoid the crowding of data points in the figure, only one set of the data for each sample is plotted, obtained during the cooling process. Here, by presenting a linear fitting of the curve, Δ*S* is obtained from the slope of the fitting line. In Figure 8a the Δ*V*-Δ*T* curves are almost linear, and Δ*S* ranges from 0.11 μV/K to 0.75 μV/K. The one measured from the 0.70 mm–0.12 mm combination shows the largest Δ*S,* of 0.75 μV/K, and at Δ*T* = 350 K, Δ*V* reaches 270 μV.

Similarly, Figure 8b presents a typical result measured from two Ti wires, where the thick one is 0.6 mm in diameter, and the thin one is 0.2 mm in diameter. The slope is about 0.33 μV/K. It shows a change of sign at Δ*T* ≈ 50 K, and exhibits a roughly linear behavior at higher Δ*T*.

## 4. Discussion

From our analysis of the measurement results for the control samples, we concluded that the system noise level and measurement error of the whole setup was less than 1 μV. All of the measured data from various type-U samples are much higher than this level. They should not be measurement errors. Yet, the dimensions of the thick and thin arms of each sample examined in this work, are three to four orders of magnitude higher than the critical lengths of either the electrons (*l*_e_) or phonons (*l*_p_) in the corresponding bulks. 

Is there any contribution from the contact at the cold ends? The Cu wires are used for the input of the Keithley 2182 Nanovoltmeter, which could induce a small voltage at the contact region, as most of the sample materials are different from Cu. However, during the measurement process, both the cold ends were carefully kept at the same temperature; the room temperature. According to the nature of the *Seebeck effect*, such a configuration may introduce a shift of voltage at Δ*T* = 0 K, but it should not contribute to the rest of the measurement results.

The repeatedly observed phenomenon in the W, Mo, Cu, brass, and Zn samples, where Δ*V* roughly increases with Δ*T* in linear fashion, indicates an unambiguous size effect. Generally, this size effect becomes stronger when the cross-sectional area ratio of the thick arm to the thin arm, increases. The results are consistent with the similar effects observed in thin film stripes of 10 metals (i.e., Bi, Cr, Ni, Sc, Ti, Pd, Pt, Ta, W, and Zr) [19,40].

**Figure 9:**
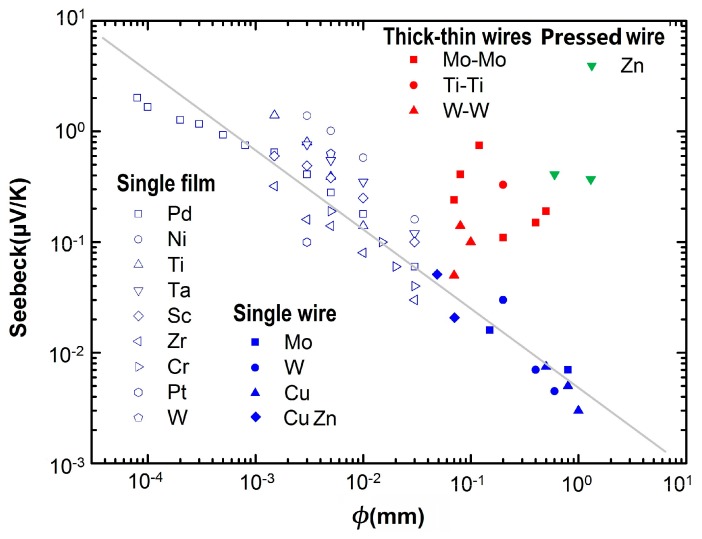
An overall view of the experimental data of a variety metallic samples. Those obtained from a single piece of thin film are plotted in hollow blue symbols. Those obtained from a single piece of thick wire are plotted in solid blue symbols. The data obtained from thick-wire, thin-wire dual-arm samples are plotted in solid red symbols. Data for the pressed Zn wire are plotted in solid green symbols. *φ* is a nominal dimension.

In Figure 9, we plot the experimental data measured from a variety of metallic samples. The data obtained from dual-stripe samples, each being fabricated with a single piece of thin film, are plotted in hollow blue symbols. The data obtained from Type-U samples of this work, each being made with a single piece of thick wire, are plotted in solid blue symbols. The data obtained from thick-wire, thin-wire dual-arm samples in this work, each being made with two wires of the same material but different diameters, are plotted in solid red symbols. Moreover, the additional data for the pressed Zn wire are plotted in solid green symbols. Here, the dimension coordinate *φ* is defined in the following: For those dual-stripe thin film samples, the width of the narrower stripe is taken as the *φ* value; for Type-U and dual-arm wire samples, the thickness of the grinded arm, or the thinner wire diameter, is taken as the *φ*. 

All of the samples made from single piece of material (either thin film or wire) share a similar trend: when *φ* reduces, the absolute value of its thermopower also reduces, and Δ*S* increases when *φ* decreases. Indeed, as shown with the grey line in the figure, one may roughly see a power law of Δ*S* ∝ *φ*^−*α*^, where *α* ~ 1.0. This resembles the observations of the thermoelectric properties of quasi-1D nanomaterials, such as nanotubes, nanowires, and nanobelts, where the size effect was manifested in the diameter dependence of the materials [46,47,48].

We may give a brief discussion on the origin of this size effect. The *Seebeck effect* is a complicated effect. Among metals, some have positive *S* values, whilst some have negative ones; and the absolute *S* values have a range of 0–20 μV/K. In most metals, the *S* value also changes with *T*. Therefore, *S* is sensitive to the electronic structure, such as the density of the state near the Fermi level. It is also sensitive to the complex electron-electron and electron-phonon scattering processes; therefore, it is sensitive to impurities, defects, the micro-structure, and the surface roughness of the material. Each of these factors may play a role in the present results. To obtain a clear quantitative correlation for the size effect of the *Seebeck coefficient* in thick wires, more systematical experiment data is required. Generally, in Equation (1), the term ${\left(d\ln A/dE\right)}_{E=E_{F}}$ is considered approximately constant, when the dimension or size of a material is much larger than the mean free path. In addition to the carrier density and temperature, the factors that determine the mean free path are mainly various scattering mechanisms between the charge carriers and material lattices, including background scattering, surface scattering, and grain boundary scattering [49,50,51]. The background scattering mainly involves the impurities and defects in the atomic structure of a material; therefore, is not very relative to the boundary conditions of the material. Surface scattering and grain boundary scattering significantly contribute to the change in the mean free path, and thus, they may be the main factors that determine the size effect of the thermopower. 

However, the size effect might not be the only mechanism that accounted for all of the results reported in this paper. For the thick-wire, thin-wire dual arm samples, and for the pressed Zn wire samples, as shown in Figure 9, much larger Δ*S* values have been observed. In these cases, the measured difference may also result from other factors, such as impurities, defects, different crystalline structures, etc., which exist in the two arms of each sample being tested. For example, although the data sheet for the 0.07 mm diameter Mo wire is the same as that of the 0.7 mm diameter Mo wire, the exact impurity level in these two wires could be different enough to induce an obvious change in Δ*S*. If such a change is controllable and stable at high Δ*T*, it is good for the application of these dual-wire devices as high-temperature sensors. 

## 5. Conclusions

In summary, by reducing the cross-section of one arm by mechanically grinding or splitting, we have made “Type-U” samples from individual pieces of bulky metallic wires of W, Mo, Cu, Ti, Cu, brass, and Zn, with diameters which range from 0.5 mm to 2.0 mm. Such a sample structure avoided the influence of the interface at the junction region of a conventional thermocouple device, and also avoided the influence of the difference in chemical components and impurities. As a result, we measured the “intrinsic” change in thermopower Δ*S* (Seebeck coefficient), which was as small as 1–10 nV/K in a metallic wire, when its cross-sectional area changes. This Δ*S* is roughly in the order of 1/1000 to 1/10,000 of the thermopower of the related bulk. This small geometric effect in bulky metallic wires seems to have been overlooked in previous studies. 

The result may have a potential application for measuring ultrahigh temperatures. However, the underlying mechanism is still not clear. Modification of current theories may be required to explain the effect. 

## Figures and Tables

**Table 1:** The main ingredients of wire materials used in this work.

Wire Material	W	Mo	Ti	Zn	Cu	Brass	
Main ingredient (greater than or equal to) **%**	99.95	99.95	99.9	92.66	99.9	Cu	Zn
						63.58	36.4
